# Lightweight Explicit 3D Human Digitization via Normal Integration

**DOI:** 10.3390/s25051513

**Published:** 2025-02-28

**Authors:** Jiaxuan Liu, Jingyi Wu, Ruiyang Jing, Han Yu, Jing Liu, Liang Song

**Affiliations:** 1 Academy for Engineering and Technology, Fudan University, Shanghai 200433, China; jiaxuanliu22@m.fudan.edu.cn (J.L.); jingyiwu23@m.fudan.edu.cn (J.W.); ryjing22@m.fudan.edu.cn (R.J.);; 2 Innovation Platform for Academicians of Hainan Province, Haikou 570228, China

**Keywords:** three-dimensional human reconstruction, normal map estimation, a skinned multi-person linear model, deep learning

## Abstract

In recent years, generating 3D human models from images has gained significant attention in 3D human reconstruction. However, deploying large neural network models in practical applications remains challenging, particularly on resource-constrained edge devices. This problem is primarily because large neural network models require significantly higher computational power, which imposes greater demands on hardware capabilities and inference time. To address this issue, we can optimize the network architecture to reduce the number of model parameters, thereby alleviating the heavy reliance on hardware resources. We propose a lightweight and efficient 3D human reconstruction model that balances reconstruction accuracy and computational cost. Specifically, our model integrates Dilated Convolutions and the Cross-Covariance Attention mechanism into its architecture to construct a lightweight generative network. This design effectively captures multi-scale information while significantly reducing model complexity. Additionally, we introduce an innovative loss function tailored to the geometric properties of normal maps. This loss function provides a more accurate measure of surface reconstruction quality and enhances the overall reconstruction performance. Experimental results show that, compared with existing methods, our approach reduces the number of training parameters by approximately 80% while maintaining the generated model’s quality.

## 1. Introduction

Recent advancements in deep learning and computer vision have significantly heightened interest in 3D reconstruction, which has emerged as a critical technological enabler across diverse domains. 3D reconstruction involves utilizing software and hardware technologies to digitally reconstruct scenes, objects, or individuals in three dimensions. Compared with 2D data, 3D information inherently encapsulates more comprehensive and precise spatial characteristics, enabling 3D models to deliver richer contextual insights than conventional 2D imagery. This capability allows computers to preserve and perceive the three-dimensional world better, transcending the limitations of 2D data representation. Currently, 3D reconstruction has been widely adopted in fields such as autonomous driving, 3D hologram generation and reconstruction [1], 3D shape reconstruction [2], and structure-from-motion photogrammetry [3], empowering innovative applications across industries.

With the evolution of 3D reconstruction techniques, 3D human reconstruction has progressively gained prominence. Recent developments have achieved remarkable progress, demonstrating substantial practical value and potential, thereby enabling broad applications in areas such as virtual reality (VR), human–computer interaction (HCI) [4], healthcare [5], 3D feature extraction [6], sports analytics [7], and cultural heritage conservation [8].

The widespread application potential of 3D human reconstruction across multiple domains has made the efficient reconstruction of human models from images a critical research direction in computer vision [9,10] and computer graphics [11]. However, achieving high-fidelity reconstruction remains challenging due to the human body’s complex geometric structures, appearances, and articulated nature. In recent years, significant progress has been made in non-parametric reconstruction methods. PIFu (Pixel-Aligned Implicit Function for High-Resolution Clothed Human Digitization) [12] and PIFuHD (Multi-Level Pixel-Aligned Implicit Function for High-Resolution 3D Human Digitization) [13] leverage implicit functions and utilize scanned 3D human data as supervision to achieve detailed reconstruction of clothed humans. However, they do not incorporate structural priors of the human body. Subsequently, PaMIR (Parametric Model-Conditioned Implicit Representation for Image-Based Human Reconstruction) [14] introduced the SMPL model (A Skinned Multi-Person Linear Model) [15] as a prior, significantly improving pose estimation [16] accuracy and overall reconstruction precision. However, it relies solely on implicit functions for representation, lacking more refined reconstruction techniques. Parametric models such as SMPL and SMPL-X have been widely adopted in virtual reality, game development, and virtual try-on systems. Compared with the nude human models reconstructed by SMPL and SMPL-X, our method achieves more detail and can reconstruct complex clothing.

With the growing demand for realistic human models across various fields, many methods have proposed innovative architectures and training techniques to enhance reconstruction performance. However, challenges persist, particularly the limitations of large-scale network models in practical applications. The core focus of this study is to reconstruct high-precision 3D human models from a single 2D image, emphasizing normal map estimation and reconstruction. Additionally, human pose estimation [16] and texture generation techniques are employed. While occlusion handling and dynamic scene reconstruction are significant challenges in 3D modeling, these aspects fall outside the scope of this work. We utilize the SMPL-X model as a prior for human pose estimation. By adopting the concept of normal map estimation, we generate front and back normal maps of the human body. The surface 3D information is represented by the normal maps computed by the generative network. To efficiently generate human surface normal maps, we design a lightweight and efficient generative network architecture. This architecture leverages the expanded receptive fields of Dilated Convolutions [17] and the cross-channel feature extraction capabilities of Cross-Covariance Attention [18]. During this process, the front and back of the human body are processed separately. The generated normal maps are then used to recover the mesh model through the d-BiNI (depth-aware silhouette-consistent bilateral normal integration) method [19], combined with the SMPL-X model (Expressive Body Capture: 3D Hands, Face, and Body from a Single Image) [20] to complete the mesh reconstruction. During training, we introduce a surface folds loss function [21] to generate more detailed fold variations and a spatial loss function [22] to measure the distances between points in 3D space. These two loss functions jointly constrain the normal map generation network, ensuring the network optimizes in the correct direction.

**Figure 1:**
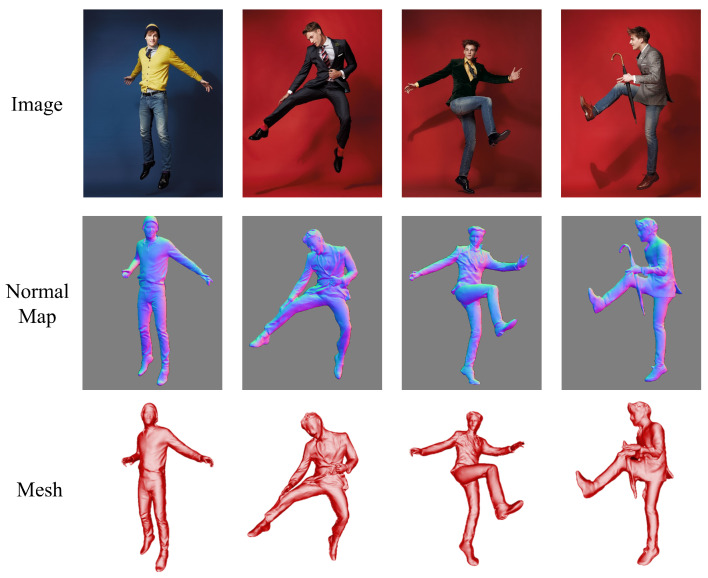
This figure demonstrates our method’s reconstruction capability. Our approach successfully generates detailed 3D human models. The top row shows the input images, the middle row presents the generated normal maps, and the bottom row displays the reconstructed 3D human models.

Figure 1 presents the reconstruction results of different human models. To reduce the parameter count of the generative network, we combine Dilated Convolutions with a Cross-Covariance Attention mechanism to build a lightweight network. Furthermore, we propose an innovative loss function to evaluate normal maps better. The final results demonstrate a balance between high reconstruction quality and model efficiency. Our contributions include the following:

- We propose an innovative human reconstruction method incorporating an innovative loss function designed to optimize the training process. This loss function significantly enhances the accuracy and detail of reconstructions, as evidenced by the promising results achieved in our experiments;
- We also introduce a lightweight generative network designed to produce high-quality surface normal maps of the human body. This network employs efficient architectural designs to capture intricate geometric details effectively. Experimental results confirm the robustness and efficiency of our approach in generating accurate and realistic surface normal maps;
- Extensive experiments were conducted to evaluate our proposed method comprehensively. These assessments, comprising both quantitative and qualitative analyses, underscore the superiority of our approach. The results demonstrate consistent performance across diverse scenarios, highlighting its robustness and effectiveness in reconstructing detailed human models.

The structure of this paper is organized as follows: Section 1 systematically elaborates on the research background, problem definition, and the academic significance of this work. Section 2 reviews the latest research progress in related fields, focusing on analyzing the strengths and limitations of existing methods. Section 3 provides a detailed description of the proposed algorithmic framework and network architecture design. Section 4 thoroughly investigates the effectiveness and performance of the proposed method through systematic experimental validation and comparative analysis. Finally, Section 5 summarizes the main innovative contributions of this study, objectively discusses the current limitations, and outlines potential directions for future research.

## 2. Related Works

In 3D human reconstruction, mainstream approaches can be categorized into “parametric” and “non-parametric” methods. Parametric methods focus on reconstructing human models by leveraging statistical human models. These methods can be further divided into those that learn body shape and pose-related corrections through statistical techniques [23,24] and those that address non-linear deformations and compensate for artifacts caused by linear blend skinning [25,26]. Parametric reconstruction relies on low-dimensional vectors to represent body shapes. This approach accurately reconstructs human shape and pose while reducing data representation complexity. Standard parametric models include SCAPE (SCAPE: shape completion and animation of people) [23], SMPL [15], and SMPL-X [20]. These models infer human parameters through optimization or regression methods. For example, SMPLify (Keep It SMPL: Automatic Estimation of 3D Human Pose and Shape from a Single Image) [27] optimizes SMPL parameters (including shape and pose) by minimizing the reprojection error between detected 2D key points and a synthesized 3D pose while incorporating penetration constraints to reduce 2D-to-3D ambiguity. HMR (end-to-end recovery of human shape and pose) [28] introduces the reprojection error of human joints into the loss function to supervise SMPL pose and shape parameters. Inspired by Generative Adversarial Networks (GANs) [29], HMR also incorporates a discriminator into the loss function to ensure the validity of predicted human parameters. SPIN (Learning to Reconstruct 3D Human Pose and Shape via Model-fitting in the Loop) [30] combines regression- and optimization-based approaches for 3D pose and shape estimation. It uses a regression network to generate SMPL parameters as initial values for an iterative fitting module. The optimization results of the iterative fitting module are then used as supervision to improve the regression network, enabling cyclic training for enhanced generation.

Non-parametric methods primarily focus on generating detailed surface models of clothed human bodies. To accommodate clothed human shapes, numerous approaches [12,19] achieve precise clothing modeling by adjusting mesh vertices. Implicit representations have also been widely adopted in non-parametric 3D reconstruction. For instance, PIFu [12] introduces pixel-aligned implicit reconstruction, while PIFuHD [13] enhances geometric details through a multi-level architecture and normal maps predicted from RGB images. However, these methods do not incorporate structural priors of the human body, leading to suboptimal reconstruction results. GeoPIFu (GeoPIFu: geometry and pixel-aligned implicit functions for single-view human reconstruction) [31] incorporates rough volumetric human shapes and methods like Self-Portrait [32], PINA (Pina: Learning a personalized implicit neural avatar from a single rgb-d video sequence) [33], and S3 (S3: Neural Shape, Skeleton, and Skinning Fields for 3D Human Modeling) [34] utilize depth or LiDAR data to regularize shapes and improve robustness to pose variations. PaMIR [14] and DeepMultiCap [35] align pixel-aligned features on posed voxelized SMPL meshes, using parametric models as priors to ensure completeness and stability. However, their reconstruction results are often coarse and unsatisfactory in many cases. ARCH (ARCH: Animatable Reconstruction of Clothed Humans) [36], ARCH++ (ARCH++: Animation-Ready Clothed Human Reconstruction Revisited) [37], and CAR (High-fidelity clothed avatar reconstruction from a single image) [38] use SMPL to map pixel-aligned query points from the posed space to a canonical space. ICON (ICON: Implicit Clothed Humans Obtained from Normals) [39] extends this to unseen poses in in-the-wild images through local feature regression, achieving impressive reconstruction quality. However, it underutilizes the generated normal maps and suffers from a large model size. ECON (ECON: Explicit Clothed Humans Optimized via Normal Integration) [19] introduces the d-BiNI technique and adopts ResNet [40] as the backbone for its generative network, achieving promising results. Nevertheless, there remains room for optimization regarding model size and inference speed.

In generative networks, an effective architecture design is crucial. R-MSFM (R-MSFM: Recurrent Multi-Scale Feature Modulation for Monocular Depth Estimating) [41] proposes a compact feature modulation module to learn multi-scale features, achieving competitive performance using only the first three stages of ResNet-18 as the backbone. Zhang et al. [42] propose a hybrid architecture combining convolutions and transformers, effectively reducing the model size while maintaining performance. Bae et al. [43] enhance CNN features with Transformers to achieve state-of-the-art accuracy, but using multiple parallel modules slows down processing speed. ICON and ECON employ ResNet [40] as the backbone for their normal map generation networks, achieving high-quality results. However, their large model sizes and slow inference speeds limit their practicality.

## 3. Methods

### 3.1. Human Models Reconstruction

Generating a 3D human model from a single image is challenging, often resulting in abnormal outputs. To address this, we incorporate a parametric human model (SMPL-X) [20] as prior knowledge to ensure human pose accuracy. This approach has been effectively utilized in methods such as PaMIR [14], ICON [39], and ECON [19]. By estimating the pose from the input image and generating a corresponding SMPL-X model [20], we infer the surface normal map of the human body based on the input image and the SMPL-X model to produce accurate normal maps.

**Figure 2:**
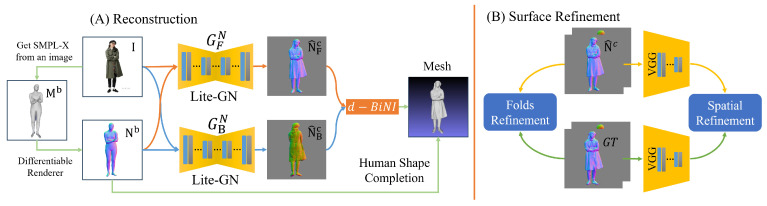
Overview. (**A**) Pipeline for 3D human reconstruction; (**B**) architectural framework of the loss function for normal map generation.

**Figure 3:**
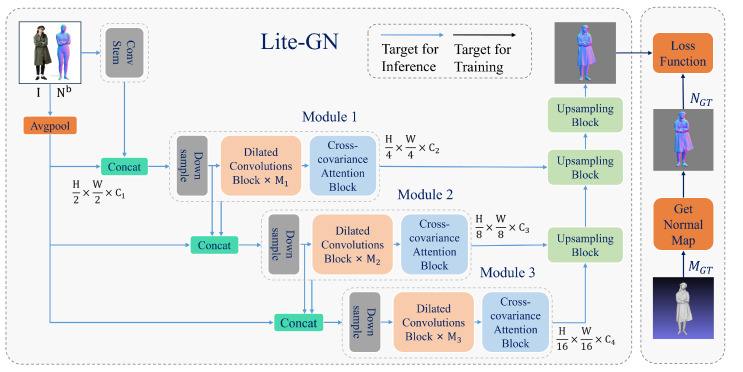
Overview of Lite-GN. The generative network is designed using an encoder–decoder architecture. Each module comprises $M_{N}$ Dilated Convolution Blocks and a Cross-Covariance Attention Block.

**Figure 4:**
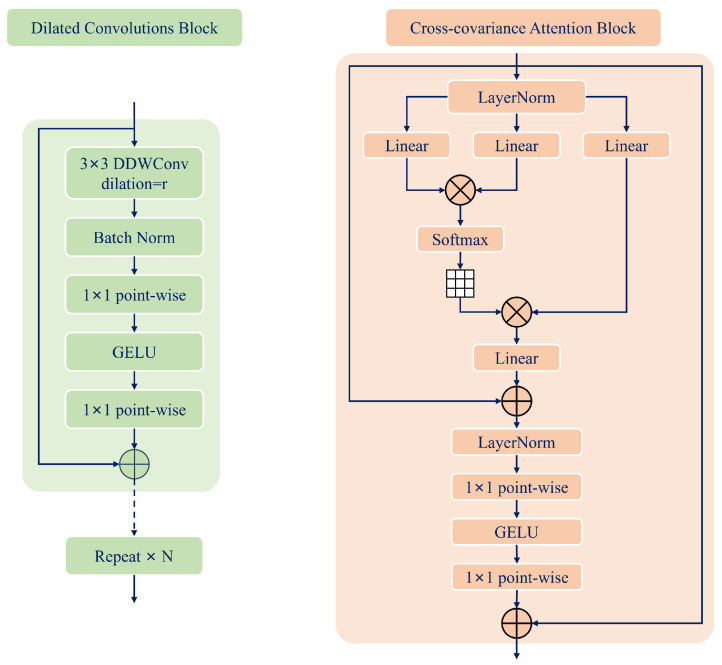
The detailed architectures of the Dilated Convolution Block and Cross-Covariance Attention Block are illustrated.

As illustrated in Figure 2A, we first perform SMPL-X model estimation on the reconstructed image and utilize a Differentiable Renderer to generate the surface normal map $N^{b}$ from the reconstructed SMPL-X model $M^{b}$. Subsequently, the image and the normal map are fed into two independent Lite-GN networks to produce front-view and back-view normal maps. These outputs are then integrated through the d-BiNI method and a human shape completion stage to reconstruct a complete 3D human model. Lite-GN primarily consists of a Dilated Convolutions Block and a Cross-Covariance Attention Block. The detailed architecture of Lite-GN is illustrated in Figure 3, while the specific structures of the Dilated Convolutions Block and the Cross-Covariance Attention Block are shown in Figure 4. To refine the surface geometry during training, we jointly optimize two refinement loss terms (as illustrated in Figure 2B)—namely, the fold refinement loss and the spatial refinement loss—by comparing the predicted normal maps with their ground truth. This approach effectively minimizes surface irregularities and geometric discrepancies.

We first perform pose estimation on the input image and generate an SMPL-X model in the corresponding pose. Our approach generates clothed human models based on the SMPL-X model to reduce ambiguity and guide the prediction of the clothed body from both the front and back perspectives. Using PyTorch3D’s (version 0.7.1, developed by Facebook AI Research, Menlo Park, CA, USA) differentiable renderer (denoted as DR), we extract surface normal maps for the SMPL-X model from both front and back views, as illustrated in Figure 2. The input image and the normal maps ${\hat{N}}^{b}=\left\{{\hat{N}}_{F}^{b},{\hat{N}}_{B}^{b}\right\}$ are then fed into the generative network $LiteG^{N}=\left\{G_{F}^{N},G_{B}^{N}\right\}$, which outputs the normal maps of the human body for the front and back views, denoted as ${\hat{N}}^{c}=\left\{{\hat{N}}_{F}^{c},{\hat{N}}_{B}^{c}\right\}$.

(1) $$DR\left(M^{b}\right)=N^{b},$$

(2) $$LiteG^{N}\left(N^{b},I\right)={\hat{N}}^{c},$$

where $M^{b}$ refers to the SMPL-X model generated based on pose parameters, and ${\hat{N}}^{c}=\left\{{\hat{N}}_{F}^{c},{\hat{N}}_{B}^{c}\right\}$ is the predicted normal map generated by $G^{N}$.

We employ a depth-aware silhouette-consistent bilateral normal integration method (d-BiNI) for the generated normal maps. This technique utilizes the prior information from the SMPL-X model. It enforces consistency along the silhouette to ensure alignment between high-frequency surface details and the predicted dense normal maps. At the same time, it maintains low-frequency surface variations, including discontinuities, by the SMPL-X model. The objective function [19] is composed of five primary components:

(3) $$d\text{-}BiNI\left({\hat{N}}_{F}^{c},{\hat{N}}_{B}^{c},Z_{F}^{b},Z_{B}^{b}\right)\to {\hat{Z}}_{F}^{c},{\hat{Z}}_{B}^{c},$$

(4) $$\begin{array}{cc} \min_{{\hat{Z}}_{F}^{c},{\hat{Z}}_{B}^{c}} & L_{n}\left({\hat{Z}}_{F}^{c},{\hat{N}}_{F}^{c}\right)+L_{n}\left({\hat{Z}}_{B}^{c},{\hat{N}}_{B}^{c}\right)+\lambda_{d}L_{d}\left({\hat{Z}}_{F}^{c},Z_{F}^{b}\right)+\\ & \lambda_{d}L_{d}\left({\hat{Z}}_{B}^{c},Z_{B}^{b}\right)+\lambda_{s}L_{s}\left({\hat{Z}}_{F}^{c},{\hat{Z}}_{B}^{c}\right), \end{array}$$

where $L_{n}$ is the BiNI [44] loss term, $L_{d}$ is the prior depth of the front and back depth surfaces, $L_{s}$ is the front and back silhouette consistency term, and $Z_{b}^{*}$ and ${\hat{Z}}_{*}^{c}$ represent the coarse body depth image and the clothed body depth image. The detailed calculation formula for $L_{d}\left({\hat{Z}}_{i}^{c},Z_{i}^{b}\right)$ and $L_{s}\left({\hat{Z}}_{F}^{c},{\hat{Z}}_{B}^{c}\right)$ [19] are as follows:

(5) $$L_{d}\left({\hat{Z}}_{i}^{c},Z_{i}^{b}\right)={\left|{\hat{Z}}_{i}^{c}-Z_{i}^{b}\right|}_{\Omega_{n}\cap \Omega_{z}}\;i\in \left\{F,B\right\},$$

(6) $$L_{s}\left({\hat{Z}}_{F}^{c},{\hat{Z}}_{B}^{c}\right)={\left|{\hat{Z}}_{F}^{c}-{\hat{Z}}_{B}^{c}\right|}_{\partial \Omega_{n}},$$

where $\Omega_{n}$ and $\Omega_{z}$ represent the domains of the clothed and body regions, and $\partial \Omega_{n}$ denotes the silhouette of $\Omega_{n}$.

**Refining SMPL-X:** Our approach utilizes the SMPL-X model as prior information; the accuracy of pose parameters extracted from images is critical for achieving precise reconstruction results. Accurate parameters provide more reliable priors, thereby enhancing the quality of the generated outcomes. To this end, we employ the PIXIE method [45] to extract SMPL-X parameters. However, the parameters directly inferred from the human model often fail to fully align with the poses and shapes depicted in the input images. To address this limitation, we further refine the SMPL-X model parameters through an optimization process [30,39]. Specifically, we optimize the shape parameter β, pose parameter θ, and translation parameter *t* of the SMPL-X model to ensure that the generated model closely aligns with the poses and appearances of the input images. The objective of this optimization process is to minimize the following loss function [19]:

(7) $$L_{SMPL-X}=\min_{\theta ,\beta ,t}\left(\lambda_{N\_diff}L_{N\_diff}+L_{S\_diff}\right),$$

(8) $$L_{N\_diff}=\left|N^{b}-{\hat{N}}^{c}\right|,\;\;\;\;\;\;\;\;\;L_{S\_diff}=\left|S^{b}-{\hat{S}}^{c}\right|,$$

where $L_{N\_diff}$ represents the normal map loss (L1), weighted by $\lambda_{N\_diff}$, and $L_{S\_diff}$ is the L1 loss measuring the difference between the silhouette of the SMPL body normal map $S_{b}$ and the human mask $S_{bc}$, which is segmented from *I* as described in Rembg [46].

**Human shape completion:** We adopt the method of ECON-EX [19,47] to complete the surface reconstruction of the human body, using the SMPL-X model to fill in the missing surface. We remove triangles of the generated mesh from the SMPL-X model $M^{b}$. The remaining triangles include side view boundaries and occluded areas, $M^{r}$. We obtain a complete human body model by using PSR [47] to merge the generated surface with the remaining surface.

### 3.2. The Proposed Framework: Lite-GN

Several studies have shown that a well-designed encoder can extract more effective features, leading to improved generative results [48,49]. We designed a lightweight generative network that efficiently generates normal maps from input images. The network consists of an encoder (Encoder Net) and a decoder (Decoder Net), as illustrated in Figure 3. To reduce the model size effectively, we utilize stacked Dilated Convolutions [17] to decrease the network depth while maintaining a large receptive field. Additionally, we incorporate a Transformer [50] to learn global features and employ Cross-Covariance Attention [18] to focus on inter-channel relationships. This architect significantly reduces the computational complexity.

**Encoder:** We achieve efficient feature encoding by introducing multi-scale information fusion. The process is as follows: First, the input human image is processed through four 3×3 convolutional downsampling layers to extract local features, resulting in a feature map of size 256×256×64. In the Module 1 stage, the downsampled feature map is concatenated with the pooled input image. It is followed by two 3×3 convolutional downsampling operations, producing a feature map of size 128×128×64. The features are then passed through a Dilated Convolutions Block to expand the receptive field, a process repeated six times. The resulting features are subsequently fed into a Cross-Covariance Attention Block to incorporate cross-covariance mechanisms. In the Module 2 stage, the input feature map is processed through two 3×3 convolutional operations, producing a feature map of size 64×64×128. The features are then passed through a Dilated Convolutions Block, repeated six times, and subsequently into a Cross-Covariance Attention Block. In the Module 3 stage, the input feature map undergoes two 3×3 convolutional operations, generating a feature map of size 32×32×224. These features are then processed through a Dilated Convolutions Block, repeated 18 times, followed by another Cross-Covariance Attention Block. It marks the completion of the encoding phase. As shown in Table 1, [Conv3×3] indicates using a 3×3 convolutional kernel.

**Decoder:** We adopt a decoder adapted from monodepth [48]. As illustrated in Figure 3, we employ multiple bilinear upsampling layers for effective upsampling, while convolutional layers connect features from the three stages of the encoder. This design ensures deep fusion of high-level and low-level features, preserving low-dimensional information while minimizing information loss. The generated normal map is output at the topmost layer.

#### 3.2.1. Dilated Convolutions Block

We employ multiple layers of Dilated Convolutions to extract features from the input image and the SMPL-X normal maps. Stacking several dilated convolution layers enhances feature integration and fusion.

For a given input feature x[i], the resulting output feature y[i] produced by a dilated convolution [17] can be expressed as follows:

(9) $$y\left[v\right]=\sum_{u=1}^{U}x\left[v+r\cdot u\right]w\left[u\right],$$

where w[u] denotes a filter of size *U*, and *r* specifies the dilation rate applied to the input x[v] during the convolution operation. In the case of standard convolution (without dilation), the dilation rate *r* is set to 1.

By employing dilated convolution, the network can maintain the size of the output feature map while achieving an expanded receptive field. The output of our Dilated Convolutions Block is as follows:

(10) $$\hat{X}=Linear\left(G\left(Linear\left(BN\left(DDWConv_{r}\left(X\right)\right)\right)\right)\right)+X,$$

where Linear refers to a point-wise convolution, and *G* represents the GELU [51] activation function. BN denotes a batch normalization layer, while $DDWConv_{r}\left(X\right)$ refers to a 3×3 depthwise dilated convolution with a dilation rate of *r*.

#### 3.2.2. Cross-Covariance Attention Block

In the Cross-Covariance Attention Block, the input feature *X* is processed through point-wise convolutions to generate queries $Q=XW_{q}$, keys $K=XW_{k}$, and values $V=XW_{v}$, where $W_{q}$, $W_{k}$, and $W_{v}$ are weight matrices. Cross-Covariance Attention [18] efficiently handles high-resolution images by combining the precision of traditional Transformers with the scalability of convolutional architectures. This approach effectively reduces the quadratic complexity of attention computation to linear complexity:

(11) $$\tilde{X}=X+Attention\left(Q,K,V\right),$$

where $Attention\left(Q,K,V\right)=V\cdot Softmax\left(Q^{T}\cdot K\right)$ [18].

Additionally, we enhance the non-linearity of the features as follows:

(12) $$\hat{X}=X+Linear\left(G\left(Linear\left(LN\left(\tilde{X}\right)\right)\right)\right),$$

where LN denotes the layer normalization [52] operation, Linear represents a point-wise convolution, and *G* is the GELU [53] activation function.

### 3.3. Surface Refinement

The VGG Loss [21] enhances the perceptual quality of generated images by performing comparisons in feature space, making it particularly suitable for tasks requiring high fidelity and structural consistency. Instead of directly comparing image differences in pixel space, VGG Loss computes the loss using intermediate feature representations extracted from a pre-trained VGG network [21]:

(13) $$L_{VGG}=\frac{1}{h_{l}w_{l}c_{l}}\sqrt{\sum_{i,j,k}{\left(\phi_{i,j,k}^{\left(l\right)}\left({\hat{N}}^{c}\right)-\phi_{i,j,k}^{\left(l\right)}\left(N_{gt}\right)\right)}^{2}},$$

where $h_{l}$, $w_{l}$, and $c_{l}$ represent the feature map’s height, width, and number of channels at layer *l*, respectively. The network is denoted as ϕ, and the high-level representation extracted at layer *l* is $\phi^{\left(l\right)}\left(N\right)$. The Euclidean distance is computed between the high-level representations of $N_{gt}$ and ${\hat{N}}^{c}$ at the corresponding layer.

Thus, the VGG network’s feature representations are closer to the human visual system’s understanding of images. This allows it to better capture high-level semantic information and perceptual quality, making it more effective in reflecting normal maps’ semantic information and quality.

The VGG Loss focuses solely on perceptual differences but disregards spatial information. To address this, we employ Huber Loss [22] to measure the spatial discrepancy between two normal maps. The Huber Loss addresses the limitations of the VGG loss by directly comparing the differences between two binary vectors at each position, precisely capturing spatial information. It is because the VGG loss, based on feature maps extracted from a pre-trained network, primarily focuses on perceptual similarity (e.g., textures and styles) in images but overlooks the geometric alignment of vertices in 3D space. In contrast, the Huber Loss quantifies spatial inconsistencies by counting the number of differing elements at corresponding positions in the two vectors, forcing the model to prioritize geometric accuracy during optimization. The formula for Huber Loss [22] is as follows:

(14) $$L_{\delta }\left(N_{gt},{\hat{N}}^{c}\right)=\left\{\begin{array}{cc} \frac{1}{2}{\left(N_{gt}-{\hat{N}}^{c}\right)}^{2}, & \left|N_{gt}-{\hat{N}}^{c}\right|\leq \delta \\ \delta \left|N_{gt}-{\hat{N}}^{c}\right|-\frac{1}{2}\delta^{2}, & \left|N_{gt}-{\hat{N}}^{c}\right|>\delta \end{array},\right.$$

where δ represents a hyperparameter that determines the balance between MSE and MAE. As a result, Huber Loss combines the advantages of both MSE and MAE.

So, we train the Generate network, Lite-GN, with the following loss:

(15) $$L_{N}=L_{\delta }\left(N_{gt},{\hat{N}}^{c}\right)+\lambda_{VGG}L_{VGG},$$

where $\lambda_{VGG}$ denotes the weight of the VGG loss.

During the training phase (as illustrated in Figure 3), we extract the front and back normal maps $N_{gt}$ from the ground truth mesh $M_{gt}$ corresponding to the input image. These $N_{gt}$ maps, along with the generated normal maps $\hat{N_{c}}$, are fed into the loss function $L_{n}$ to train the normal map generation network, LiteGN. The specific details of the loss function are illustrated in Figure 2B and described by Equation (15).

## 4. Experiments and Results

### 4.1. Implementation Details

**Datasets:** We utilize the THuman2.0 dataset [54] as our training set to train the generative network. THuman2.0 consists of 525 high-quality human scan models and has been used to train methods such as ECON [19], ICON [39], IF-Nets (Implicit Function Networks) [19], PIFu [12], and PaMIR [14]. Additionally, we use the CAPE [55] and Renderpeople datasets [56] for quantitative evaluation. The CAPE dataset assesses the robustness of reconstruction methods under complex poses, while the Renderpeople dataset evaluates robustness in handling intricate clothing.

**Figure 5:**
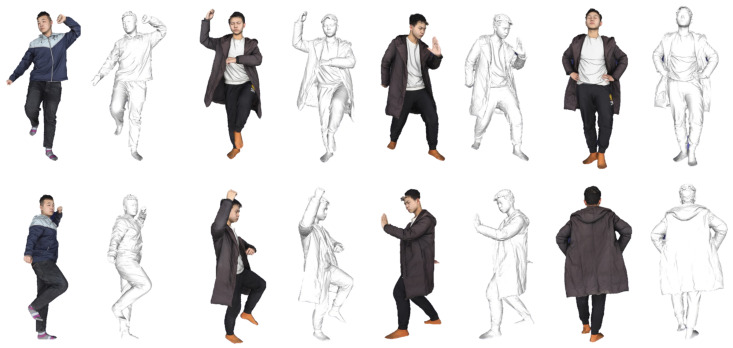
Examples from the THuman2.0 dataset.

The THuman2.0 dataset is acquired using high-precision scanners, providing high-resolution textured 3D human models. Each model is accompanied by corresponding SMPL and SMPL-X parameters for the respective poses (as illustrated in Figure 5).

**Training stage:** We use PIXIE (Collaborative regression of expressive bodies using moderation) [45] to extract SMPL-X parameters from the input images, which are then utilized to construct the SMPL-X model. The AdamW optimizer is employed with an initial learning rate of $10^{-3}$, which is reduced by a factor of 0.1 every 10 epochs. The training process spans 50 epochs with a batch size of 4. Detailed experimental environment parameters are shown in Table 2.

### 4.2. Evaluation Metrics

**Chamfer distance:** Chamfer distance [57] is a widely used metric in 3D geometry processing to evaluate the similarity between two point sets. Precisely, it measures the average squared distance from each point in the ground truth scan to its nearest neighbor in the generated model. This symmetric calculation ensures that both point sets are equally accounted for in the evaluation. Chamfer distance is particularly effective for assessing the overall geometric alignment between the ground truth and the reconstructed model, making it a reliable indicator of the quality of the generated result. A lower Chamfer distance suggests the reconstructed model closely approximates the actual geometry.

**P2S distance:** Point-to-Surface (P2S) distance [47] is a more refined metric that measures the nearest distance from each point in the ground truth scan to the closest surface in the reconstructed model. Unlike Chamfer distance, which treats both datasets as point clouds, P2S distance incorporates the reconstructed model’s surface information. It makes it especially valuable for evaluating how well the reconstructed surface conforms to the underlying geometry of the ground truth. By focusing on the proximity between points and surfaces, P2S distance provides a nuanced understanding of reconstruction accuracy, particularly in areas where precise surface details are critical.

**Normals difference:** Normals difference [19] assesses the angular difference between the normal vectors at corresponding points in the ground truth and reconstructed models. Normal vectors encode the local orientation of surfaces and are essential for capturing fine geometric details and high-frequency structures. By comparing these vectors, normal differences highlight discrepancies in local surface characteristics that may not be evident from positional metrics alone. This metric is crucial for evaluating the preservation of intricate details, such as sharp edges and subtle surface undulations, in the reconstruction process. Smaller differences in normals indicate a higher fidelity reconstruction of local geometric features.

### 4.3. Evaluation

#### 4.3.1. Quantitative Evaluation

Our method enables the reconstruction of detailed 3D human models from a single image. It can generate complete human models for individuals of any gender or role. Even in complex poses and clothing cases, our approach maintains high reconstruction quality, as illustrated in Figure 1. The method accurately reconstructs clothing and poses while preserving critical facial features.

We conducted a comprehensive comparison of our method with several state-of-the-art approaches, including ECON [19], ICON [39], PaMIR [14], and PIFuHD [13]. To thoroughly evaluate the performance of each method, we employed four key metrics: Chamfer Distance, Point-to-Surface (P2S) Distance, Normals Difference, and Weight Size, which collectively measure the superiority of the approaches. The results of the comparison are presented in Table 3. For a fair evaluation, all methods were trained using the THuman2.0 dataset [54], which provides high-quality 3D human scans, and tested on the CAPE dataset [55], a challenging benchmark that captures diverse clothing variations. The experimental results demonstrate that our method achieves generation quality comparable to PaMIR and ICON regarding visual fidelity and structural accuracy, ensuring highly competitive results. Although some metrics are slightly inferior to ECON-EX, our approach significantly reduces the model size, highlighting its superior lightweight characteristics. As shown in Table 4, our method achieves more outstanding results regarding lightweight efficiency.

We also performed a focused comparison with other methods that utilize normal maps for 3D reconstruction. In this comparison, we evaluated the performance of the normal map generation networks across three key metrics: the number of network parameters, the size of the model weight files, and the runtime during inference. As shown in Table 4, our method substantially reduces resource consumption. Specifically, our approach minimizes network parameters and weight file size while significantly shortening the runtime, enabling faster processing and reduced computational overhead. This efficient resource utilization highlights the practical advantages of our method, especially in scenarios where computational resources are constrained or real-time performance is critical. By balancing quality and efficiency, our approach is well suited for applications requiring lightweight 3D human reconstruction.

Additionally, we evaluated our approach on the Renderpeople dataset to test its robustness to various types of clothing under complex apparel conditions. We also adopted Chamfer Distance, P2S Distance, and Normals Difference as key evaluation metrics. The results indicate that our method achieves high-quality generation outcomes on the RenderPeople dataset, which is consistent with its performance on the CAPE dataset. While the generation quality surpasses most comparative methods, it is slightly inferior to ECON-EX, as shown in Table 5. However, our approach significantly reduces the model size, demonstrating its superior lightweight efficiency.

#### 4.3.2. Qualitative Comparison

**Figure 6:**
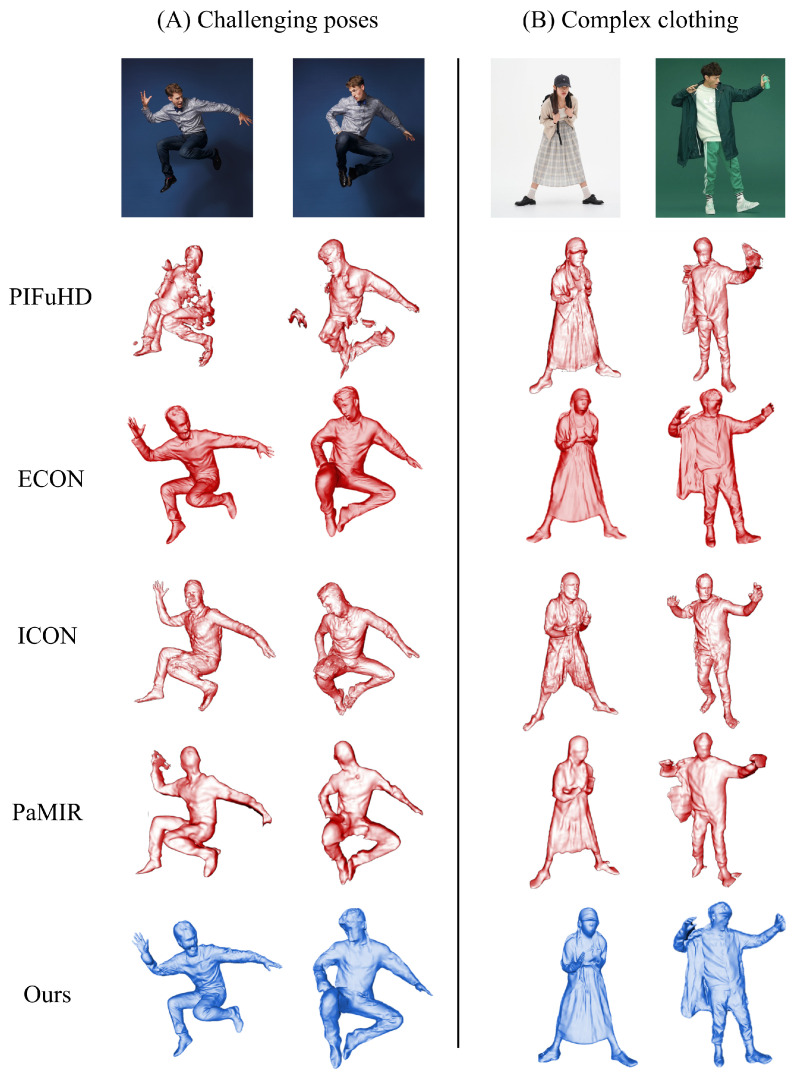
Existing methods exhibit varying limitations in 3D human reconstruction: PIFuHD [13] demonstrates competent clothing reconstruction capabilities but encounters challenges with complex pose estimation. While ICON [39] achieves reasonable overall performance, its ability to reconstruct intricate clothing details remains constrained. Similarly, PaMIR [14] shows insufficient reconstruction fidelity, particularly in capturing fine-grained surface details. In contrast, both ECON [19] and our proposed method demonstrate superior reconstruction quality, effectively addressing these limitations through advanced architectural designs.

We compared our method against other human reconstruction approaches. The test images were divided into two “complex poses” and “complex clothing”. As shown in Figure 6, our method effectively reconstructs the visual characteristics of the human body. The first row presents the input images, serving as the input for monocular reconstruction. On the left are images with complex human poses, showcasing our method’s capability to handle challenging poses. On the right are examples of intricate clothing, highlighting the robustness of our approach to complex apparel. The qualitative comparison results align with the quantitative findings in Table 3. The results demonstrate that our method significantly outperforms PIFuHD [13] and PaMIR [14] while achieving comparable performance to ECON [19] and ICON [39], with the added advantage of a more lightweight network.

**Figure 7:**
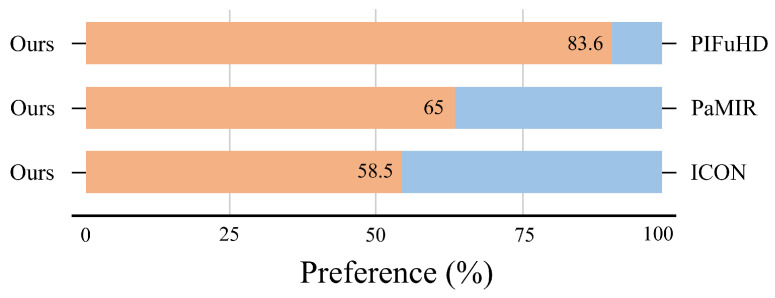
User preference. Our method demonstrated higher user preference compared with the baseline approach. The user study results further validate our approach’s effectiveness in meeting the demand for high-quality human body reconstruction. In the figure, the orange color represents our method, while the blue color indicates the comparative method.

**User preference:** To complement our quantitative and qualitative evaluations, we conducted a user study to assess participants’ subjective preferences regarding reconstruction results based on the CAPE dataset. In this study, participants were presented with reconstruction results from two methods: our proposed approach and a comparative method. They were instructed to select the reconstruction they perceived as superior in quality, considering aspects such as pose accuracy, clothing details, and the precision of facial and hand features. Data were collected from 43 participants, each evaluating 10 pairs of reconstructed human models for each comparison. As shown in Figure 7, our method was consistently preferred over the comparative method, demonstrating its superiority.

### 4.4. Ablation Study

To further validate the effectiveness of the proposed model, we conducted a series of ablation studies to evaluate the importance and contribution of different modules and designs in our method. Specifically, we systematically removed or modified certain key modules in the network and analyzed the impact of these changes on model performance. The experiments were conducted on the CAPE dataset [55], which features diverse pose variations, making it suitable for testing the model’s performance under complex postural conditions. By comparing the experimental results under different configurations (as shown in Table 6), we analyzed the specific contributions of each module to the quality of generated results, runtime efficiency, and overall model performance.

**Benefits of the Dilated Convolutions Block:** To evaluate the impact of Dilated Convolutions, we conducted an ablation study by setting the dilation rate of all Dilated Convolutions in the Dilated Convolutions Block to 1, turning off the dilation mechanism. Experimental results showed a decrease in accuracy, indicating that incorporating the Dilated Convolutions Block expands the receptive field, addressing the limitations of shallow networks with constrained receptive fields, as shown in Table 6.

**Benefits of the Cross-Covariance Attention Block:** To assess the role of the Cross-Covariance Attention Block, we performed experiments by removing all instances of this block. Results demonstrated a decline in accuracy, confirming that the Cross-Covariance Attention Block is essential for extracting cross-channel feature information. This mechanism compensates for the limitations of CNNs, which extract local features.

**Benefits of VGG Loss:** The ablation studies conducted on our network architecture revealed a noticeable reduction in reconstruction accuracy when VGG Loss was excluded. It underscores the critical role of VGG Loss in enhancing the performance of normal map generation. By capturing high-level semantic information and improving perceptual quality, VGG Loss ensures that the generated normal maps align closely with human visual perception. Its ability to emphasize finer details and contextual relationships significantly contributes to the overall quality and fidelity of the reconstructions, making it an indispensable component of our loss function design.

**Benefits of Huber Loss:** The experimental results demonstrated a decline in accuracy when Huber Loss was omitted from the training process, indicating its crucial contribution to the network’s performance. Huber Loss excels in balancing the sensitivity to outliers while maintaining stability during optimization. By penalizing large deviations less aggressively than traditional L1 or L2 losses, it enables the network to extract spatial information effectively. This loss function leads to improved robustness and precision in handling variations in geometric structures, particularly in areas with subtle transitions or intricate details. Its inclusion ensures the generation of accurate and realistic surface normal maps, further enhancing the overall reconstruction quality.

### 4.5. Facial and Hand Refinement Attempts

**Figure 8:**
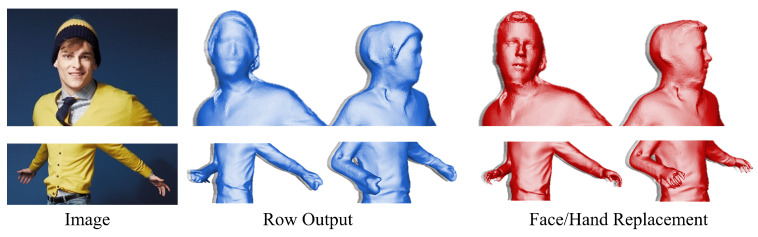
To enhance the reconstruction quality, the facial and hand regions of the generated model are replaced with the corresponding components from the SMPL-X model.

We further explored enhancing the facial and hand details of the reconstructed characters by replacing their original features with those generated using the SMPL-X face model. This approach was designed to achieve greater precision and refinement in facial features and hand articulation. The results, illustrated in Figure 8, reveal that this replacement significantly enhances the overall realism and intricacy of facial expressions and hand details. However, it also introduces a notable trade-off: the characters’ unique and distinctive traits are diminished, leading to a loss of individuality. This finding highlights the challenge of balancing detail and realism with preserving personalized attributes in human reconstruction tasks.

### 4.6. Text-Driven Texture Generation

We draw inspiration from the texture method proposed by TEXTure [58] to generate surface textures for human models. Utilizing a pre-trained depth-to-image diffusion model [59] guided by textual prompts, we render the 3D model from various viewpoints. Subsequently, we employ a trimap partitioning of the rendered images into three progression states. This process leverages the trimap representation to create seamless textures from different views, ultimately completing the mapping for the entire model.

**Figure 9:**
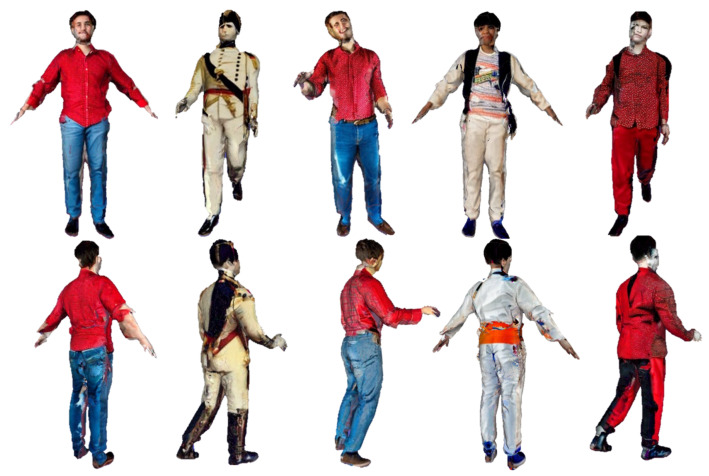
This figure demonstrates the generated texture maps from various viewpoints using different text prompts.

By providing different textual descriptions, such as “Napoleon”, “a 30-year-old man wearing a red shirt, blue pants, and brown shoes”, or “an 18-year-old boy wearing a red jacket, red pants, and black shoes”, we generated various results, as shown in the Figure 9, and obtained outstanding experimental results.

### 4.7. Animatable Avatar Creation

**Figure 10:**
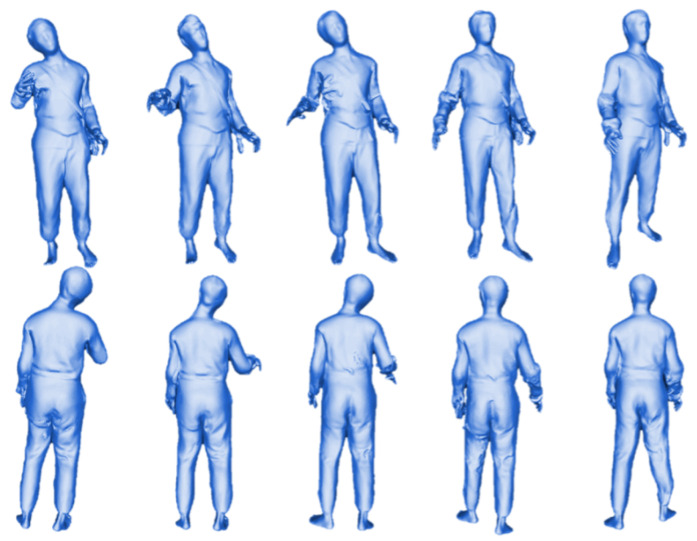
We developed a pose-parameter-driven avatar using generated human models based on the SCANimate [60] framework.

We further evaluated the animatability of the 3D human models generated by our method. Specifically, we reconstructed a series of images depicting the same individual in various poses and input the resulting 3D models and their corresponding SMPL models into SCANimate [60]. By leveraging our generated results, SCANimate effectively learns and represents the deformations of human models under different poses. This process significantly facilitates the construction of animatable avatars by providing high-quality input data, enabling better capture of complex geometric variations in human bodies. As shown in Figure 10, the experimental results demonstrate that our method contributes to generating high-quality animatable avatars, highlighting the effectiveness of our reconstructed outputs.

## 5. Conclusions

This paper introduces an innovative framework for 3D human reconstruction, achieving significant advancements in accuracy, efficiency, and realism. Our approach leverages SMPL-X as a prior to generating surface normal maps, which are subsequently integrated to reconstruct high-quality 3D human models. We introduce a custom loss function to refine the quality of the models, while SMPL-X priors are utilized to recover missing details. Additionally, we employ a diffusion model to generate surface textures and utilize SCANimate to produce an animatable avatar. To further enhance efficiency, we propose a lightweight normal map generation network incorporating Dilated Convolutions and Cross-Covariance Attention mechanisms, significantly reducing computational complexity without compromising performance. Experimental results demonstrate that our method achieves superior reconstruction quality while optimizing network size and parameters. However, challenges remain in handling complex poses and clothing, where arteficts such as missing limbs or inconsistent back-side reconstructions may occur. Future work will focus on improving robustness to extreme poses, refining clothing reconstruction, and enhancing overall consistency, with the aim of advancing monocular 3D human reconstruction for broader real-world applications.

## Figures and Tables

**Table 1:** The network parameters of the Encoder.

Output Size	Stage	Layer
512 × 512 × 6	Input	
256 × 256 × 64	Conv Stem	[Conv3 × 3] × 4
		[Conv3 × 3] × 2
128 × 128 × 64	Stage 1	Dilated Convolutions Block × 6
		Cross-Covariance Attention Block × 1
		[Conv3 × 3] × 2
64 × 64 × 128	Stage 2	Dilated Convolutions Block × 6
		Cross-Covariance Attention Block × 1
		[Conv3 × 3] × 2
32 × 32 × 224	Stage 3	Dilated Convolutions Block × 18
		Cross-Covariance Attention Block × 1

**Table 2:** Experimental environment’s parameters.

Equipment	Computer Configuration Parameters
Operating system	Linux
RAM	32 G
Type of operating system	Ubuntu20.04
CPU	Intel Core i7-12700K
GPU	RTX 4090(24 GB) × 1
Development language	Python 3.8
Deep learning framework	PyTorch 1.12.1

**Table 3:** Quantitative comparison on CAPE dataset.

Methods	Chamfer ↓	P2S ↓	Normals ↓	Weight Size ↓
PIFuHD [13]	3.767	3.591	0.0994	1.4 GB
PaMIR [14]	0.989	0.992	0.0422	472 MB
ICON [39]	0.971	0.909	0.0409	1.38 GB
ECON-IF [19]	0.996	0.967	0.0413	1.49 GB
ECON-EX [19]	0.926	0.917	0.0367	1.35 GB
Ours	0.965	0.930	0.0472	201.6 MB

↓ indicates that lower values are better.

**Table 4:** Model complexity and speed evaluation. We evaluated and compared the parameters, model weight file size, and inference speed of different methods.

Methods	Model Size ↓	Weight Size ↓	Speed (ms) ↓
ICON [39]	345.6 M	1.35 GB	22.8 ms
ECON [19]	345.6 M	1.35 GB	23.6 ms
Ours	50 M	201.6 MB	12.8 ms

↓ indicates that lower values are better.

**Table 5:** Quantitative comparison on Renderpeople dataset.

Methods	Chamfer ↓	P2S ↓	Normals ↓
PIFuHD [13]	1.946	1.983	0.0658
PaMIR [14]	1.296	1.430	0.0518
ICON [39]	1.373	1.522	0.0566
ECON-IF [19]	1.401	1.422	0.0516
ECON-EX [19]	1.342	1.458	0.0478
Ours	1.353	1.430	0.0452

↓ indicates that lower values are better.

**Table 6:** Ablation study on CAPE dataset.

Methods	Chamfer	P2S	Normals
w/o Cross-Covariance Attention Block	1.074	0.994	0.0535
w/o Dilated Convolutions Block	1.139	1.087	0.0569
w/o VGG Loss	1.560	1.607	0.0662
w/o Huber Loss	1.917	1.973	0.0546

## Data Availability

The data are included in the article.
